# Supplementary material for: Genome-Wide Identification of the BnaRFS Gene Family and Functional Characterization of BnaRFS6 in Brassica napus
Source: Genes (Basel). 2025 Aug 29;16(9):1032. doi: 10.3390/genes16091032 (PMC12469805; doi:10.3390/genes16091032)
Supplement: Supplementary file 1 [file genes-16-01032-s001.zip › genes-3784948-supplementary.pdf]

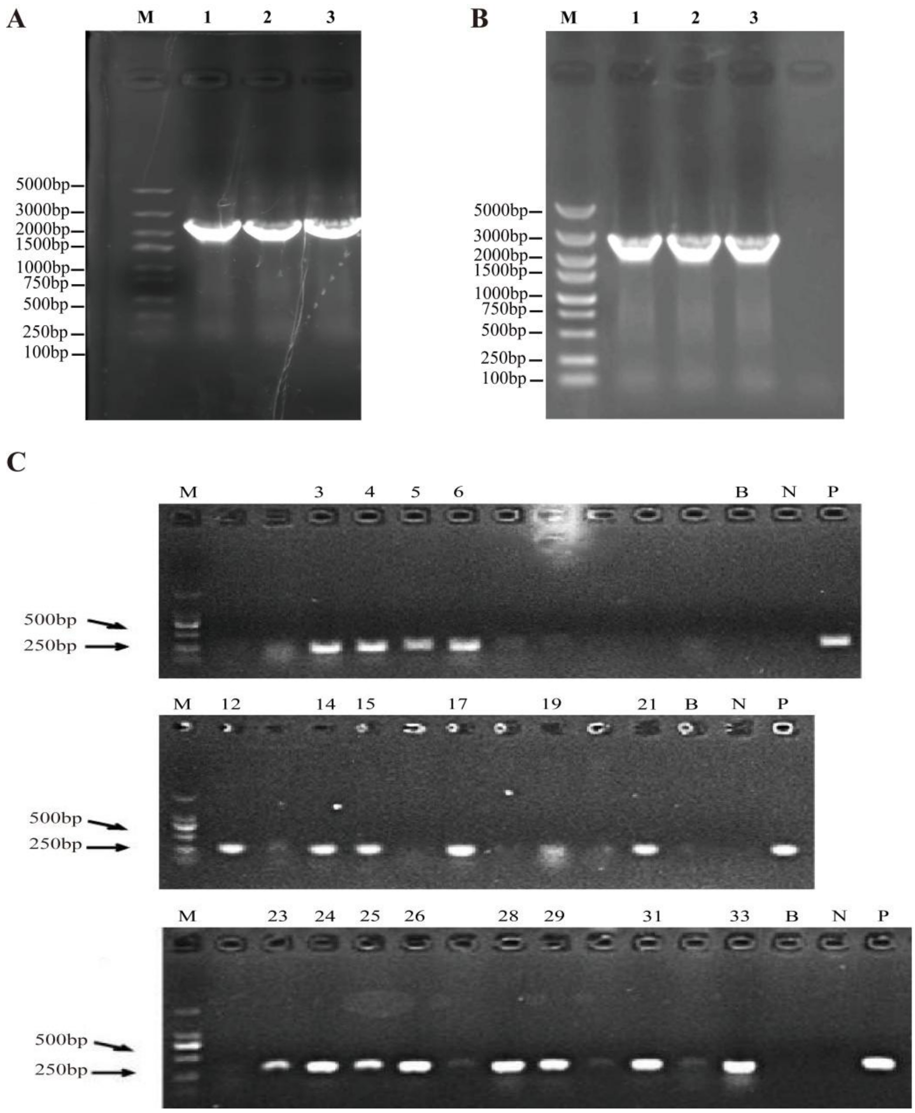

Supplementary Figure S1. Agarose gel electrophoresis. (A) Clone of *BnaRFS6*, M: DL5000 DNA marker. (B) OE-*BnaRFS6* vector PCR detection, M: DL5000 DNA marker. (C) Positive detection of OE-*BnaRFS6* plants, M: DL2000 DNA marker, B: CK, N: Negative Control, P: Positive Control.

Supplementary Table S1. Gene Primers

Primers	Primers sequences (5′-3′)	Purpose	Accession Number of Gene
BnaRFS6-F	ATGGCGTCACAGGGGTG	Clone	-
BnaRFS6-R	TCATAACTCAATTTCCATCACG		
NPTII-F68	ACTGGGCACAACAGACAATCG	Positive detection	-
NPTII-R356	GCATCAGCCATGATGGATACTTT		
RFS6-F	CTCCTGGGAAGCACAA	qRT-PCR	XP_022555614.2
RFS6-R	CGGCGAATAAACAATC		
Actin2.1-F	GGTTGGGATGGACCAGAAGG	qRT-PCR	ACS68187.1
Actin2.1-R	TCAGGAGCAATACGGAGC		
